# Factors Associated With Clinical and Radiographic Severity in People With Osteoarthritis: A Cross-Sectional Population-Based Study

**DOI:** 10.3389/fmed.2021.773417

**Published:** 2021-11-15

**Authors:** Daniela Costa, Eduardo B. Cruz, Catarina Silva, Helena Canhão, Jaime Branco, Carla Nunes, Ana M. Rodrigues

**Affiliations:** ^1^NOVA National School of Public Health, Public Health Research Centre, Universidade NOVA de Lisboa, Lisbon, Portugal; ^2^Comprehensive Health Research Centre, Universidade Nova de Lisboa, Lisbon, Portugal; ^3^EpiDoC Unit, Chronic Diseases Research Centre (CEDOC), NOVA Medical School, Universidade NOVA de Lisboa, Lisbon, Portugal; ^4^Physiotherapy Department, School of Health, Polytechnic Institute of Setúbal, Setúbal, Portugal; ^5^Rheumatology Unit, Centro Hospitalar Lisboa Ocidental (CHLO-E.P.E.), Hospital Egas Moniz, Lisbon, Portugal; ^6^Rheumatology Unit, Hospital dos Lusíadas, Lisbon, Portugal

**Keywords:** prevalence, hip osteoarthritis, knee osteoarthritis, clinical severity, radiographic severity

## Abstract

**Background:** Hip/knee osteoarthritis (HKOA) is a leading cause of disability and imposes a major socioeconomic burden. The aim of this study is to estimate the prevalence of HKOA in Portugal, characterised the clinical severity of HKOA in the population, and identified sociodemographic, lifestyle, and clinical factors associated with higher clinical and radiographic severity.

**Methods:** Participants with a diagnosis of HKOA from the EpiReumaPt study (2011–2013) were included (*n* = 1,087). Hip/knee osteoarthritis diagnosis was made through a structured evaluation by rheumatologists according to American College of Rheumatology criteria. Clinical severity was classified based on Hip Disability and Osteoarthritis Outcome Scale (HOOS) and Knee Injury and Osteoarthritis Outcome Scale (KOOS) score tertiles. Radiographic severity was classified based on the Kellgren-Lawrence grades as mild, moderate, or severe. Sociodemographic lifestyle and clinical variables, including the presence of anxiety and depression symptoms, were analysed. Factors associated with higher clinical and radiographic severity were identified using ordinal logistic regression models.

**Results:** Hip/knee osteoarthritis diagnosis was present in 14.1% of the Portuguese population [12.4% with knee osteoarthritis (OA) and 2.9% with hip OA]. Clinical severity was similar between people with hip (HOOS = 55.79 ± 20.88) and knee (KOOS = 55.33 ± 20.641) OA. People in the high HOOS/KOOS tertile tended to be older (64.39 ± 0.70 years), female (75.2%), overweight (39.0%) or obese (45.9%), and had multimorbidity (86.1%). Factors significantly associated with higher clinical severity tertile were age [55–64 years: odds ratio (OR) = 3.18; 65–74 years: OR = 3.25; ≥75 years: OR = 4.24], female sex (OR = 1.60), multimorbidity (OR = 1.75), being overweight (OR = 2.01) or obese (OR = 2.82), and having anxiety symptoms (OR = 1.83). Years of education was inversely associated with higher clinical severity. Factors significantly associated with higher radiographic severity were age (65–74 years: OR = 3.59; ≥75 years: OR = 3.05) and being in the high HOOS/KOOS tertile (OR = 4.91). Being a female and live in Lisbon or in the Centre region were inversely associated with the higher radiographic severity.

**Conclusion:** Hip/knee osteoarthritis is present in ~1.1 million of Portuguese people. Age, educational level, and obesity are independently associated with HKOA clinical severity, whereas age, sex, geographic location, and clinical severity are independently associated with radiographic severity.

## Introduction

Osteoarthritis (OA), which is the most common articular disease, is a leading cause of chronic disability and a major public health problem (1). Globally, more than 300 million people have hip and/or knee osteoarthritis (HKOA), which is responsible for 9.6 million years lived with disability, and its incidence and prevalence continue to rise (2).

Worldwide, the direct annual mean cost per patient with HKOA is estimated to be 6.7 k€, reaching 10.8 k€ if patients undergo total joint replacement (3). Moreover, the annual indirect cost per patient may surpass the direct cost and is estimated to range from 0.2 k€ to 12.3 k€ (3). Portugal has the highest growth rate of total joint replacement among Organisation for Economic Co-operation and Development countries, with a 20% increase in incidence between 2005 and 2011 (4). Additionally, indirect costs due to premature exit from work represent 0.4% of the Portuguese gross domestic product (5).

Overweight and high body mass index (BMI), physical inactivity, previous joint injuries, and ageing are the main risk factors for the onset and severity of HKOA (6). Data from the EpiReumaPt study reveals that, in the Portuguese population, female sex, higher age, multimorbidity, low levels of physical activity, and physical disability are associated with the diagnosis of OA among adults with ≥50 years old (7). Similar to other middle- and high-income countries (8), Portugal is an ageing country, in which 80% of older adults are overweight and 75% of the adult population is physically inactive (9). Therefore, the prevalence of HKOA and its associated socioeconomic burden is expected to increase exponentially over the next decades (10).

People with HKOA often experience chronic pain, fatigue, sleep problems, disability, impaired quality of life, with a consequent negative impact on mental health, which progressively limits their participation in social, leisure, and occupational activities (1, 11). People with HKOA have heterogeneous presentations and disease severity depending on factors such as structural joint damage, the presence of non-communicable diseases (e.g., diabetes, obesity), risk factor exposure, age of symptom onset, and psychosocial factors (12). Therefore, radiographic and clinical severity are important predictors of individual burden and healthcare service utilisation (13). Although there is no consensus on the gold standard for evaluating HKOA severity, the general recommendation is to use a combination of radiographic and clinical severity measures (14).

Internationally, much attention has been paid to suboptimal outcomes of HKOA at the patient and system levels, as epidemiological data raise concerns about the severity of HKOA and the escalating burden of this disease (15). However, in Portugal, there are little available epidemiological data on the severity of HKOA and its associated factors. Thus, we aimed to estimate the prevalence of HKOA in Portugal, characterise the clinical severity of HKOA in the population, and identify sociodemographic, clinical, and lifestyle factors associated with clinical and radiographic severity. This information is crucial for obtaining a better understanding of the individual burden of HKOA, estimating future increases in health resource demands, and identifying needs for implementing prevention and management strategies for people with HKOA.

## Materials and Methods

### Data Source

**Figure 1:**
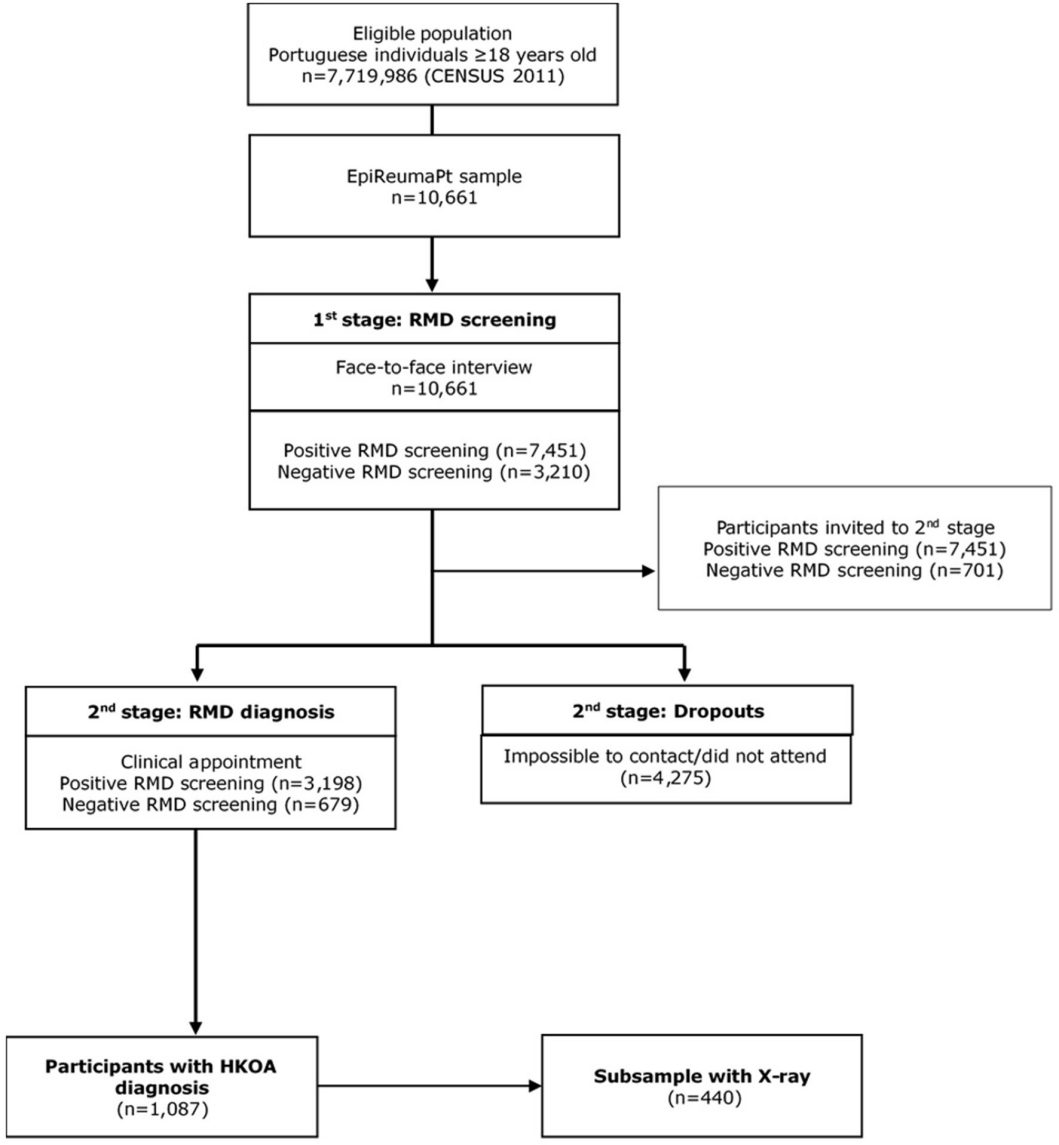
EpiReumaPt study flowchart.

This study was developed under the scope of EpireumaPt, a national cross-sectional population-based study that aims to comprehensively assess the burden of rheumatic and musculoskeletal diseases (RMDs) in Portugal. EpiReumaPt includes a representative sample of the Portuguese population that was assessed to identify and characterise the population with RMD in Portugal (16). The study included non-institutionalised adults (≥18 years old) who lived in private households in Portugal Mainland and Islands (Madeira and Azores). Recruitment was conducted between September 2011 and December 2013 through a process of multistage random sampling of private households in Portugal stratified according to administrative territorial units (NUTS II: Norte, Centro, Lisboa and Vale do Tejo, Alentejo, Algarve, Azores, and Madeira) and the size of the population within each locality. In total, 28,502 households were contacted, 8,041 individuals refused to participate, and 10,661 individuals completed the interviews (Figure 1).

**Figure 2:**
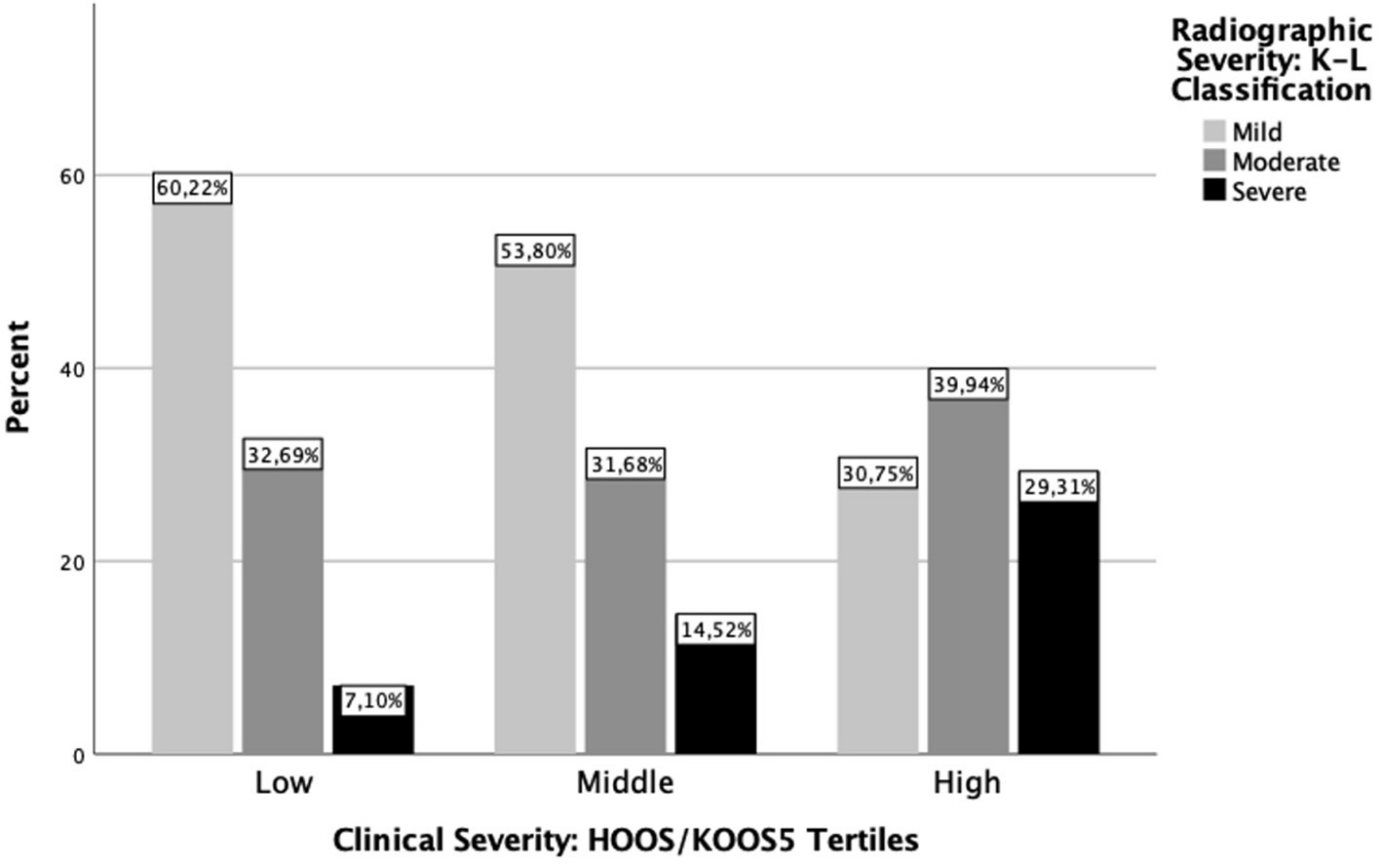
Distribution of radiographic severity classification according to clinical severity tertile. The y-axis indicates the proportion of K-L classification grades within each HOOS/KOOS tertile, and the x-axis indicates HOOS/KOOS tertile. Bars represent K-L classification grade: mild ≥2, moderate = 3, or severe = 4.

The EpireumaPt methodology consisted of a three-stage approach (Figures 2) (16). In the first stage, participants completed a face-to-face interview to collect sociodemographic and health-related information and to screen for RMDs. A person was considered to have a positive screening if they mentioned a previously known RMD, if any algorithm in the screening questionnaire was positive, or if they reported muscle, vertebral, or peripheral joint pain in the previous 4 weeks. Interviews were conducted by a team of non-medical healthcare professionals who were trained for this purpose, and data were collected using a computer-assisted personal interview system.

Participants who screened positive for at least one RMD (*n* = 7,451) and ~20% (*n* = 701) of participants with negative screening for RMDs were invited for the second stage, which consisted of a clinical appointment with a rheumatologist. Of these participants, 4,275 did not attend the clinical appointment. Therefore, at the end of stage two, there were 3,877 clinical observations; 3,198 participants received a confirmation of RMD, and 679 did not have an RMD. Clinical assessments were performed at the primary care centre within the participant's neighbourhood by a multidisciplinary team consisting of a rheumatologist, X-ray technician, and nurse. Clinical appointments consisted of a structured evaluation, laboratory, and imaging exams, if needed, to establish the diagnosis and evaluate disease-related information. Simple X-rays were performed for 122 hips and 479 knees, among other joints, according to participants' musculoskeletal complaints. The rheumatologists involved were blind to prior health-related data.

In the third stage, three rheumatologists reviewed all data and validated the diagnoses. Diagnostic agreement among the three rheumatologists was 98.3% with a Cohen's K coefficient of 0.87 [95% confidence interval (CI): 0.83, 0.91] (16). When data were insufficient to fulfil international classification criteria for each RMD, an additional meeting with the rheumatologists took place in order to reach agreement on the final diagnosis. When doubts persisted, the opinion of the rheumatologist who performed the clinical assessment in the second stage prevailed.

As the EpiReumaPt population is similar to the Portuguese population (CENSUS 2011), weights were calculated and calibrated according to age strata, sex, size of locality, and NUTS II in the first stage of the study to reproduce the known population totals for the crossing margins of these four variables. In the second stage, weights were recalibrated taking into account the inclusion probabilities considering the results of screening, and adjusted for non-response, as described in detail elsewhere (16).

### Study Population

This study included participants in the EpiReumaPt study with a validated diagnosis of HKOA according to American College of Rheumatology diagnosis criteria (17, 18).

### Outcomes

The outcomes of this study were measures of HKOA clinical and radiographic severity, which were assessed in the second stage of EpireumaPt, during clinical appointments. Clinical severity was evaluated with Portuguese versions of the Knee Injury and Osteoarthritis Outcome Scale (KOOS) (19) and Hip Disability and Osteoarthritis Outcome Scale (HOOS) (20). These self-reported outcome measures evaluate short-term and long-term consequences of HKOA in five dimensions: pain, symptoms, activities of daily living, sports and leisure, and quality of life. Scores for each dimension are transformed into a 0–100 scale, with 0 representing extreme hip/knee problems and 100 representing no hip/knee problems (19, 20). A final composite score (HOOS/KOOS) was calculated with the mean score of each dimension as previously recommended (21). Relationships between the core OA domains of pain, function, and quality of life are complex, fluctuate over time, and are intimately related to each other. Therefore, a composite score is considered optimal for capturing the multidimensional features of OA (22). We computed the tertile of the sample score distribution to categorise participants into low (65.00–100), middle (45.2–64.80), and high (0.00–45.00) tertiles of clinical severity, because there are no validated cut-offs for this measurement tool (23).

For radiographic severity, the Kellgren-Lawrence (K-L) system was used to classify joint structural deterioration using antero-posterior X-rays into four severity grades: grade 0 (normal), grade 1 [doubtful joint space narrowing (JSN) and possible osteophytic lipping], grade 2 (definite osteophytes and possible JSN on anteroposterior weight-bearing radiograph), grade 3 (multiple osteophytes, definite JSN, sclerosis, possible bony deformity), and grade 4 (large osteophytes, marked JSN, severe sclerosis, and definite bony deformity) (24). We considered the radiographic severity of HKOA as mild if K-L ≤ 2, moderate if K-L = 3, and severe if K-L = 4 (25).

For both outcome measures, if more than one joint was affected, the joint with the worse score/classification was considered.

### Sociodemographic, Clinical, and Lifestyle Factors

Sociodemographic, clinical, and lifestyle variables were collected during the first and second phase of EpiReumaPt.

#### Sociodemographic and Anthropometric Factors

Sociodemographic variables were age, sex, geographic location according to NUTS II territorial units, marital status, and education level. Madeira and Azores were merged in the analysis as the Islands region. Marital status was dichotomized as “with partner” (married or lived in consensual union) or “no partner” (single, widowed, or divorced). Education level was categorised according to years of education completed: <4 years (less than primary education), 4–9 years (primary or secondary education), or ≥10 years (secondary or superior education). Body mass index was categorised as underweight (≤ 18.49 kg/m^2^), healthy weight (≥18.5 and ≤ 24.99 kg/m^2^), overweight (≥25 and ≤ 29.99 kg/m^2^), or obese (≥30 kg/m^2^).

#### Clinical and Lifestyle Factors

Lifestyle variables included alcohol intake (“never or occasionally” or “daily” intake), smoking (“never” or “occasionally or daily”), and regular exercise/sports (“yes” or “no”).

The number of chronic non-communicable diseases was calculated as the numeric count of the following self-reported conditions: high blood pressure, high cholesterol, cardiac disease, diabetes mellitus, chronic lung disease, problems in the digestive tract, renal colic, neurological disease, allergies, mental or psychiatric illness, cancer, thyroid or parathyroid problems, hypogonadism, and hyperuricemia. Multimorbidity was defined as having ≥2 chronic non-communicable diseases (26).

The presence of anxiety and depression symptoms was evaluated using depression (HADS-D) and anxiety (HADS-A) subscales of the Hospital Anxiety and Depression Scale. Both subscales have a range from 0 to 21, with higher values representing more symptoms. Final HADS-A and HADS-D scores were categorised with validated cut-offs as “anxiety symptoms” (HADS-A ≥11) or “without anxiety symptoms” (HADS-A <11) and as “depression symptoms” (HADS-D ≥11) or “without depression symptoms” (HADS-D <11) (27).

### Data Analysis

Prevalence estimates were computed as weighted proportions for hip OA, knee OA, and HKOA. The logit transformation method was used to calculate 95% CIs.

Using descriptive statistics, participants with HKOA in each HOOS/KOOS tertile were characterised based on their sociodemographic, clinical, and lifestyle features. Mean scores on HOOS/KOOS subscales (symptoms, pain, activities of daily living, sports and leisure, and quality of life) for people with HKOA were plotted by age, sex, and radiographic severity (Supplementary Material, Supplementary Figure 1). The independency between HOOS/KOOS tertile and K-L classification was analysed using chi-square independency tests with a significance level based on adjusted F (*p* < 0.005).

Differences between HOOS/KOOS tertiles were analysed using *t*-tests for continuous variables and chi-square independency tests (*p* < 0.05) for categorical variables. As only a subsample of participants received X-rays (*n* = 440), we performed tests for independency of sociodemographic, clinical, and lifestyle characteristics between participants with and without X-rays to better interpret the final results (Supplementary Material, Supplementary Table 1).

To analyse variables associated with HOOS/KOOS tertile and K-L classification, two separate ordinal regression models were computed. During this stage of the analysis, age classes were merged as <55 years old, 55–64 years old, 65–74 years old, and ≥75 years old, and BMI categories were merged as normal or underweight (<25.00 kg/m^2^), overweight (25–29.99 kg/m^2^), and obese (≥30.00 kg/m^2^), due to low frequencies in some categories.

For each ordinal regression model, during univariate analysis, a level of significance of 0.25 for relationships between each independent variable and the outcome was considered as the cut-off to enter in the multivariable ordinal regression model, to avoid early exclusion of potentially important variables (Supplementary Material, Supplementary Table 2) (28). Sociodemographic and anthropometric, clinical, and lifestyle variables were tested in the univariate analysis. Before running the models, the assumption of proportional odds and multicollinearity were validated, and independent variables with bivariate correlations above *r* > 0.75 were excluded (29).

The logit link function (29) was used because it improved the performed of both multivariable ordinal regression models according to their classification properties and McFadden Pseudo-*R*^2^. This function computed proportional odds ratios (ORs) with 95% CIs. We used a stepwise procedure to construct the final models. Thus, in the first step, all socio-demographic and anthropometric, clinical and lifestyle variables that reached a significance level *p* < 0.25 in the univariate analysis were included. In the following steps, the variables less associated with the outcome were removed one by one, until we reached the final models, where only significant variables remained. The ordinal regression models were adjusted for sex, age, presence of multimorbidity, and BMI, which are known factors associated with HKOA severity, with potential counfounding effect. Therefore, we forced the entry of these variables in all steps. As participants with missing data were automatically excluded from this analysis, K-L classification was not included in the HOOS/KOOS model. Model fit was evaluated using McFadden Pseudo-*R*^2^. All analyses were weighted and performed with SPPS Complex Samples 26 for MacOS (IBM Corp., Armonk, NY, USA).

## Results

The weighted prevalence estimate of HKOA in Portugal was 14.1% (95% CI: 12.6, 15.7, weighted *n* = 1,138,264), with the knee being the most affected joint [knee: 12.4% (95% CI: 11.1, 13.9, weighted *n* = 1 002 192), hip: 2.9% (95% CI: 2.3, 3.7, weighted *n* = 238 038)] (Table 1). The prevalence of HKOA increased across each age class, being present in 40.9% (95% CI: 34.3, 47.8) of people ≥75 years old, and was more prevalent in women (17.5%, 95% CI: 15.3, 19.9). Clinical severity, according to HOOS/KOOS (*n* = 996), was similar between participants with hip OA and knee OA (HOOS = 55.79 ± 20.88, KOOS = 55.33 ± 20.641).

**Table 1:** Estimated prevalence of HKOA by sex, age, and severity.

	**Hip and/or Knee OA** ***n*** **= 1,087**	**Knee OA** ***n*** **= 981**	**Hip OA** ***n*** **= 199**
**Total prevalence** **% (95% CI)**	14.1 (12.6–15.7)	12.4 (11.1–13.9)	2.9 (2.3–3.7)
**Total prevalence** **weighted counts (*****n*****)**	1,138,264	1,002,192	238,038
**Prevalence by sex** **% (95% CI)**			
Male	10.4 (8.5–12.7)	8.5 (7.0–10.4)	2.9 (1.9–4.3)
Female	17.5 (15.3–19.9)	16.0 (13.9–18.2)	3.0 (2.4–3.8)
**Prevalence by age** **% (95% CI)**			
<45 years old	1.8 (1.1–2.8)	1.5 (0.4–0.9)	0.4 (0.2–0.9)
45–54 years old	14.5 (11.3–18.6)	12.0 (9.3–15.3)	3.2 (1.5–6.5)
55–64 years old	24.2 (20.0–28.9)	21.5 (17.9–25.7)	4.8 (2.6–8.6)
65–74 years old	35.5 (30.1–41.4)	31.5 (26.5–36.9)	7.4 (5.7–9.6)
≥75 years old	40.9 (34.3–47.8)	37.1 (30.8–43.8)	8.6 (11.8–23.2)
**Clinical severity**			
HOOS/KOOS score, mean ± SD	59.6 ± 21.80	55.33 ± 20.641	55.79 ± 20.88
**Radiographic severity** ***n*** **(%)**			
Mild (K-L ≤ 2)	197 (48.3)	177 (48.7)	36 (62.1)
Moderate (K-L = 3)	153 (34.0)	140 (32.1)	14 (35.8)
Severe (K-L = 4)	90 (17.8)	87 (19.2)	3 (3.2)

*All percentages and means ± standard deviations (SDs) are weighted*.

*Radiographic severity non-weighted sub-sample (n = 440): mild n = 197, moderate n = 153; severe n = 90*.

### Characterisation of Population by Clinical Severity

The mean age of the HKOA population was 64.39 ± 12.90 years old and increased from the lowest (57.82 ± 1.67 years old) to the high (64.39 ± 0.70) HOOS/KOOS tertile (Table 2). The largest proportion of the population with HKOA lived in the north of Portugal (*n* = 271, 35.8%), but clinical severity did not differ across Portugal regions. There was an unequal distribution of education levels across HOOS/KOOS tertiles; the high tertile contained the largest proportion of people with <4 years of education (*n* = 138, 38.1%) and the smallest proportion of people with ≥10 years of education (*n* = 18, 6.1%). More than 80% of people with HKOA were overweight (*n* = 387, 43.6%) or obese (*n* = 369, 35.8%) and were mostly in the middle and high HOOS/KOOS tertiles.

**Table 2:** Sociodemographic and anthropometric characteristics of participants with HKOA by clinical severity.

	**Total**	**HOOS/KOOS** **low tertile**	**HOOS/KOOS** **middle tertile**	**HOOS/KOOS** **high tertile**	***p*** **-Value^a^**
**Sample size**	*n* = 996	*n* = 281	*n* = 361	*n* = 354	
**Age (mean ± SD)**	64.39 ± 12.90	57.82 ± 1.67	66.24 ± 0.77	64.39 ± 0.70	<0.001
<45 years old, *n* (%)	37 (6.2)	32 (93.8)	3 (3.2)	2 (3.0)	<0.001
45–54 years old, *n* (%)	129 (15.6)	59 (48.1)	47 (34.3)	23 (17.6)	
55–64 years old, *n* (%)	268 (23.4)	74 (32.5)	110 (34.9)	84 (22.9)	
65–74 years old, *n* (%)	340 (31.4)	79 (26.3)	125 (35.4)	136 (38.3)	
≥75 years old, *n* (%)	222 (23.3)	37 (19.6)	76 (34.1)	109 (46.2)	
**Female**, ***n*** **(%)**	720 (65.8)	180 (51.3)	265 (71.1)	275 (75.2)	<0.001
**Geographic location**, ***n*** **(%)**					0.240
North	271 (35.8)	82 (38.6)	88 (32.2)	101 (36.4)	
Centre	243 (27.7)	58 (25.5)	86 (27.7)	99 (30)	
Lisbon	163 (23.5)	56 (25.9)	58 (24.8)	49 (19.9)	
Alentejo	67 (6.5)	9 (3.0)	32 (9.1)	26 (7.6)	
Algarve	21 (1.8)	8 (2.2)	4 (1.0)	9 (2.2)	
Islands	231 (4.7)	68 (4.8)	93 (5.3)	70 (3.9)	
**Marital status (partner)**, ***n*** **(%)**	639 (64.4)	191 (61.8)	228 (64.9)	220 (66.4)	0.664
**Years of education**, ***n*** **(%)**					<0.001
<4 years	246 (22.6)	35 (9.7)	73 (20.1)	138 (38.1)	
4–9 years	631 (62.9)	187 (67.9)	247 (65.0)	197 (55.8)	
≥10 years	117 (14.4)	58 (22.3)	41 (14.8)	18 (6.1)	
**BMI**, ***n*** **(%)**					0.003
Underweight	3 (0.2)	1 (0.4)	1 (0.1)	1 (0.3)	
Normal weight	162 (20.5)	66 (28.9)	58 (16.4)	38 (15.1)	
Overweight	387 (43.6)	134 (43.8)	139 (47.6)	114 (39.0)	
Obese	369 (35.8)	72 (26.9)	143 (35.9)	154 (45.9)	

*Categorical variables are presented as n (%); continuous variables are presented as mean ± SD. All percentages and means ± SDs are weighted*.

a *p-value from independency tests: complex samples t-tests for continuous variables and Chi-square tests for categorical variables. Significance level is based on adjusted F*.

Regarding lifestyle variables, 10.7% (*n* = 71) of Portuguese adults with HKOA smoked, and 27.8% (*n* = 211) drank alcohol daily (Table 3). Few people with HKOA performed regular physical exercise (*n* = 209, 21.3%), particularly those in the high HOOS/KOOS tertile (*n* = 53, 14.4%). The overall proportion of people with HKOA who also had multimorbidity was 74.1% (*n* = 756), which was most pronounced in the high HOOS/KOOS tertile (*n* = 305, 86.1%). The proportions of people with anxiety (*n* = 95, 26.0%) and depression (*n* = 85, 23.6%) symptoms also increased in the high HOOS/KOOS tertile.

**Table 3:** Lifestyle and clinical characteristics of participants with HKOA by clinical severity.

	**Total**	**HOOS/KOOS** **low tertile**	**HOOS/KOOS** **middle tertile**	**HOOS/KOOS** **high tertile**	***p*** **-Value^a^**
**Sample size**	*n* = 996	*n* = 281	*n* = 361	*n* = 354	
**Lifestyle variables**, ***n*** **(%)**					
Smoker	71 (10.7)	31 (17.3)	24 (9.0)	16 (5.6)	0.007
Alcohol intake (daily)	211 (27.8)	70 (33.9)	74 (26.1)	67 (23.3)	0.117
Regular exercise	209 (21.3)	81 (28.5)	75 (20.6)	53 (14.4)	0.007
**Clinical variables, mean ± SD**					
HOOS/KOOS	55.79 ± 20.88	79.50 ± 9.60	54.35 ± 5.91	33.21 ± 9.87	<0.001
(min–max)	(0.00–100)	(65.00–100)	(45.20–64.80)	(0.00–45.00)	
Multimorbidity (yes), *n* (%)	756 (74.1)	175 (60.0)	276 (76.4)	305 (86.1)	<0.001
Anxiety symptoms (HADS-A), mean ± SD	6.70 ± 4.21	6.0 ± 4.05	6.39 ± 4.18	7.72 ± 4.19	<0.001
HADS-A ≥ 11, *n* (%)	193 (18.5)	40 (12.9)	58 (16.6)	95 (26.0)	0.002
Depression symptoms (HADS-D), mean ± SD	6.04 ± 4.49	4.54 ± 4.07	5.97 ± 4.15	7.63 ± 4.68	<0.001
HADS-D ≥ 11, *n* (%)	159 (16.8)	25(11.5)	49 (15.1)	85 (23.6)	0.028

*Categorical variables are presented as n (%); continuous variables are presented as mean ± SD. All percentages and means ± SDs are weighted*.

a *p-value from independency tests: complex samples t-tests for continuous variables and Chi-square tests for categorical variables. Significance level is based on adjusted F*.

HOOS/KOOS tertiles were independent of K-L classification [*F*_(3.26;3542.35)_ = 33.69, *p*=0.002]. Across increasing HOOS/KOOS tertiles, the proportion of people with mild K-L classification decreased and the proportion of people with severe K-L classification increased (Figure 2). However, the highest clinical severity tertile was heterogeneous, consisting of 30.75% of people with mild, 39.94% of people with moderate, and 29.31% of people with severe K-L classification. Results regarding the characterisation of the population with hip and the population with knee OA are presented in Supplementary Material, Supplementary Table 2.

### Factors Associated With Clinical Severity

In the final ordinal regression model for clinical severity, the following factors were significantly associated with a higher HOOS/KOOS tertile: being 55–64 years old (OR = 3.18; 95% CI 1.80, 5.62; *p* < 0.001), 65–74 years old (OR = 3.25; 95% CI: 1.87, 5.67; *p* < 0.001), or ≥75 years old (OR = 4.24; 95% CI: 2.26, 8.00; *p* < 0.001) compared with <55 years old; being female (OR = 1.60; 95% CI: 1.09, 2.35; *p* = 0.017); having multimorbidity (OR = 1.75; 95% CI: 1.13, 2.71; *p* = 0.013); being overweight (OR = 2.01; 95% CI 1.16, 3.48; *p* = 0.013) or obese (OR = 2.82; 95% CI: 1.62, 4.90; *p* < 0.001) compared with normal weight; and having anxiety symptoms (OR = 1.83; 95% CI: 1.20, 2.81; *p* = 0.005) (Table 4). On the other hand, having 4–9 years of education (OR = 0.50; 95% CI: 0.32, 0.77; *p* = 0.002) or ≥10 years of education (OR = 0.30; 95% CI: 0.17, 0.52; *p* < 0.001) were significantly and inversely associated with a higher HOOS/KOOS tertile.

**Table 4:** Factors associated with clinical severity in the final multivariable ordinal regression model.

	**OR (95% CI)**	***p*** **-Value**
**Age**		
<55 years old^a^	–	–
55–64 years old	3.18 (1.80, 5.62)	<0.001
65–74 years old	3.25 (1.87, 5.67)	<0.001
≥75 years old	4.24 (2.26, 8.00)	<0.001
**Sex**		
Male^a^	–	–
Female	1.60 (1.09, 2.35)	0.017
**Number of non-communicable diseases**		
No multimorbidity^a^	–	–
Multimorbidity	1.75 (1.13, 2.71)	0.013
**Education level**		
<4 years^a^	–	–
4–9 years	0.50 (0.32, 0.77)	0.002
≥10 years	0.30 (0.17, 0.52)	<0.001
**BMI (kg/m** ^2^ **)**		
Normal or underweight (<25 kg/m^2^)^a^	–	–
Overweight (25–29.99 kg/m^2^)	2.01 (1.16, 3.48)	0.013
Obese (≥30 kg/m^2^)	2.82 (1.62, 4.90)	<0.001
**Anxiety (HADS-A)**		
No anxiety symptoms (HADS-A <11)^a^	–	–
Anxiety symptoms (HADS-A≥11)	1.83 (1.20, 2.81)	0.005

*Total sample included in the analysis: n = 968. Test of parallel lines: Wald F_(10)_ = 0.630; p = 0.789 (assumption of proportional odds validated); McFadden Psuedo-R^2^ = 0.115. This model correctly classified 51.4% of cases. All analysis were weighted*.

a *Reference classes*.

Compared with the results of univariate analysis, multimorbidity (OR = 2.90; 95% CI: 1.90; 4.42; *p* < 0.001) and anxiety symptoms (OR = 1.96; 95% CI: 1.33, 2.80; *p* = 0.001), and the age stratas 55–64 (OR = 3.27; 95% CI: 1.81, 5.95; *p* < 0.001) and ≥75 years old (OR = 6.06; 95% CI: 4.33, 1.00; *p* < 0.001) were less strongly associated with clinical severity in the multivariable ordinal regression model (Supplementary Material, Supplementary Table 3). By contrast, the age strata 65–74 years old (OR = 2.31; 95% CI: 2.56, 7.29) and BMI (overweight: OR = 1.87; 95% CI: 1.06, 3.30; *p* = 0.003; obese: OR = 2.72; 95% CI: 1.53, 4.85; *p* = 0.001) were more strongly associated with clinical severity in the multivariable ordinal regression model.

### Factors Associated With Radiographic Severity

Regarding the subpopulation of participants who received an X-ray (*n* = 440), the final ordinal regression model for radiographic severity showed that a severe K-L classification was associated with being 65–74 years old (OR = 3.59; 95% CI: 1.43, 9.02; *p* = 0.007) or ≥75 years old (OR = 3.05; 95% CI: 1.13, 8.21; *p* = 0.028) compared with <55 years old and being in a high HOOS/KOOS tertile (OR = 4.91; 95% CI: 2.57, 9.40; *p* < 0.001) compared with a low HOOS/KOOS tertile (Table 5). By contrast, a less severe K-L classification was associated with being female (OR = 0.41; 95% CI: 0.24, 0.69; *p* = 0.001) and living in the Lisbon (OR = 0.23; 95% CI: 0.11, 0.48; *p* < 0.001) or Centre region (OR = 0.35; 95% CI: 0.20, 0.61; *p* < 0.001).

**Table 5:** Factors associated with radiographic severity in the final multivariable ordinal regression model.

	**OR (95% CI)**	***p*** **-Value**
**Age**		
<55 years old^a^	–	–
55–64 years old	1.43 (0.54, 3.79)	0.470
65–74 years old	3.59 (1.43, 9.02)	0.007
≥75 years old	3.05 (1.13, 8.21)	0.028
**Sex**		
Male^a^	–	–
Female	0.41 (0.24, 0.69)	0.001
**Chronic non-communicable diseases**		
No multimorbidity		
Multimorbidity	0.71 (0.37, 1.36)	0.300
**Geographic location**		
North^a^	–	–
Centre	0.35 (0.20, 0.61)	<0.001
Lisbon	0.23 (0.11, 0.48)	<0.001
Alentejo	0.64 (0.30, 1.35)	0.237
Algarve	1.40 (0.27, 7.34)	0.689
Islands	0.80 (0.24, 2.65)	0.717
**BMI (kg/m** ^2^ **)**		
Normal or underweight (<25 kg/m^2^)^a^	–	–
Overweight (25–29.99 kg/m^2^)	1.61 (0.75, 3.44)	0.222
Obese (≥30 kg/m^2^)	1.67 (0.77, 3.58)	0.191
**HOOS/KOOS**		
Low tertile^a^	–	–
Middle tertile	1.69 (0.85, 3.40)	0.137
High tertile	4.91 (2.57, 9.40)	<0.001

*Total sample included in the analysis: n = 376. Test of parallel lines: Wald F_(14)_ = 0.789, p = 0.681 (assumption of proportional odds validated); McFadden Pseudo-R^2^ = 0.132. This model correctly classified 54.1% of cases. All analyses were weighted*.

a *Reference classes*.

Compared with the results of univariate analysis, the following variables were more strongly associated with radiographic severity in the multivariable ordinal regression model: age (65–74 years old: OR = 2.60; 95% CI: 1.18, 5.71; *p* = 0.018; ≥75 years old: OR = 2.71; 95% CI: 1.16, 6.35; *p* = 0.022), sex (female: OR = 0.65; 95% CI: 0.42, 1.00; *p* = 0.052), and high HOOS/KOOS tertile (OR = 3.68; 95% CI: 1.82, 7.43; *p* < 0.001) (Supplementary Material, Supplementary Table 4). By contrast, geographic location (Centre: OR = 0.49; 95% CI: 0.29, 0.83, *p* = 0.008; Lisbon: OR = 0.30; 95% CI: 0.16, 0.57; *p* < 0.001) was less strongly associated with radiographic severity in the multivariable ordinal regression model.

## Discussion

This study shows that 14.1% of people in Portugal have HKOA, mostly involving the knee, which corresponds to the ~1.1 million people with this disease in Portugal, as previously reported (11). The Portuguese dataset used in Global Burden of Diseases report (GBD) to estimate global prevalence of OA was EpireumaPt, but no data were published on HKOA together in Portugal (11). The prevalence of OA, globally, ranges from 5.4 to 24.2% for the knee and 0.9 to 7.4% for the hip (30). According to the GBD report, Portugal, the United Kingdom, and Finland have the highest age-standardised prevalence of HKOA in Europe (4,000–4,400 per 1,000,000 individuals) (2).

Different from the present study, the cohorts in several previous studies were limited to radiographic-only diagnoses or older age classes (2, 31, 32), which may not encompass people in the early stages of the disease or with early-onset HKOA. For example, the GBD report only included people with HKOA confirmed radiologically with grades 2–4 K-L and, as such, likely underestimates the true prevalence (2, 32). Our data show that the prevalence of HKOA is higher in females and increases across each age class, being present in up to 40% of people who are ≥75 years old, consistent with previous reports (2, 31, 33, 34). Although sex differences in the incidence and prevalence of HKOA have been previously studied, they are not yet fully understood. However, early exposure to oestrogen (i.e., menarche at a younger age), parity, and menopause may provide hormonal and biomechanical explanations for the greater prevalence of OA in females (35). Furthermore, we found that risk factors for the onset and severity of OA are highly prevalent in the Portuguese population, particularly the lack of regular exercise and the presence of overweight or obesity, and are even higher than those previously reported for the overall Portuguese population (9), and compared with international HKOA cohorts (36).

Although the proportion of people with moderate and severe radiographic OA increased with greater clinical severity, we found that people in the high clinical severity tertile had heterogeneous radiographic severity classifications. This finding is supported by previous studies showing that HKOA radiographic severity is correlated with clinical severity but in a non-linear fashion (14) and is an imprecise guide for predicting clinical severity (37).

Regarding radiographic severity, >80% of people in our study had mild or moderate severity, similar to other studies (38). The Chingford Women's study, a 15-year longitudinal cohort study, concluded that 41.5% of knees worsened by at least one K-L grade over this time span, with a 3.9% annual rate of disease worsening (39). Thus, if the prevalence of HKOA is increasing (10), a higher proportion of people with mild and moderate HKOA will progress to moderate or severe HKOA at a considerable rate. People with severe HKOA are 5.3 times more likely to have surgery (13), thus increasing the demand for healthcare resources and the socio-economic impact of this disease (3).

Regarding the factors associated with HKOA severity, age was associated with both clinical and radiographic severity. All older age strata were associated with a high clinical severity tertile compared with the <55 years old stratum, whereas only age strata above 65 years old were associated with high radiographic severity. These results are consistent with the observed impact of pain and functional impairment due to HKOA on social and work activities, which can lead to absenteeism, presentism, or premature work withdrawal and negatively affect the quality of life of adults at younger ages living with HKOA (5, 40). The association of clinical and radiographic OA severity with age is well-documented in the literature (2, 31, 33, 34) and is explained by both cumulative exposure to risk factors including chronic non-communicable diseases and by biological age-related structural joint changes (41). However, we found contradictory results pertaining to the association between sex and HKOA severity. Being female was simultaneously associated with higher clinical severity and milder radiographic severity. Previous literature also reports conflicting associations between sex and the clinical severity of OA (42). Data from the European SHARE cohort reveal that women have overall disadvantages in terms of activity limitations, pain, depression, and self-reported health status compared with men (43). On the other hand, a systematic review by Bastick et al. found strong evidence that sex is not associated with radiographic severity (44). However, male sex is often linked with physically demanding jobs (e.g., firefighting, construction, mining, carpentry) and contact sports, which increases the probability of previous trauma, injuries, and structural joint damage and represents a major joint-level risk factor for OA (22).

We also found that multimorbidity was associated with greater clinical severity. Non-communicable diseases are prevalent in the HKOA population and are associated with greater utilisation of healthcare services (45). A systematic review and meta-analysis by Calders et al. likewise found that a higher non-communicable disease count is associated with the worsening of pain and performance-based physical function. Specifically, the presence of cardiac disease, hypertension, and back pain are important predictors of the deterioration of physical functioning, and diabetes is associated with worse pain (46). In addition, being overweight or obese was associated with greater clinical severity but not radiographic severity in the present study. Similarly, previous research indicates that BMI is a dose-responsive risk factor for OA clinical severity (47). However, other studies report that BMI is associated with both clinical and radiographic severity (48) and that greater mechanical load (49) and systemic inflammation due to a high BMI may play a role in OA onset and clinical and radiographic severity (50).

In addition, we found that anxiety symptoms and socioeconomic factors, such as low education, were associated with clinical severity but not radiographic severity. As previously described, anxiety is associated with worse pain and physical function trajectories (51). Moreover, previous research indicates that education is an important social determinant for several chronic diseases, including OA, and is related to lifestyle factors, a lack of preventive measures, low access to healthcare services, and low literacy levels (42, 52). Psychosocial variables such as a low level of self-efficacy, catastrophising, and pain sensitisation are also associated with poorer clinical outcomes (41). Thus, the lived experience of people with HKOA and its multifactorial influences, such as psychosocial factors, may be distinct from its structural changes, suggesting that HKOA is best framed with a biopsychosocial approach (41).

This study also revealed geographic associations, with people living in the North region having higher radiographic severity than people living in the Lisbon and Centre regions. In the northern region of Portugal, industrial employment is higher than the national average, and agriculture, forestry, construction, and manufacturing industries are important sources of employment (53). A recent systematic review concludes that people in the agriculture, construction, and metal industries have a higher probability of developing knee OA (54). Additionally, due to current sociodemographic characteristics of this region and the projected prevalence of chronic diseases and long-term disability, the northern region of Portugal is expected have a higher proportion of individuals with at least one chronic disease and long-term disability in 2031, mainly due to lower education levels (55).

### Limitations

This study has some limitations that should be considered. Its cross-sectional design does not allow the establishment of a temporal relationship between associated factors and HKOA severity, thus it is not possible to establish cause-and-effect relationships between modifiable variables, such as BMI, and HKOA symptoms and physical function. The estimation of prevalence using sample weights is not free from error, although it is considered that weights should be used in all statistics analysis when dealing with complex survey data (56).

Although hip and knee OA may impose similar burdens on the domains of one's life (2, 31), some studies show that people with hip OA have greater disease severity and an earlier requirement for joint replacement (13). However, we did not thoroughly investigate differences in factors associated with hip vs. knee OA.

As HOOS and KOOS scores do not have validated cut-off values, we categorised our sample by tertile distribution. Thus, it should be noted that the low, middle, and high HOOS/KOOS tertiles do not directly correspond to mild, moderate, and severe radiographic severity. Moreover, we did not use any imputation method, given the amount of missing X-ray data, for the overall sample. The subgroup of participants who received X-rays was older, presented higher clinical severity, and had a larger number of non-communicable diseases (Supplementary Material, Supplementary Table 1); thus, this should be considered when interpreting the results.

“Regular exercise” was self-reported by participants and did not consider the precise amount and intensity of weekly physical activity. Hence, our data may overestimate the proportion of people who performed exercise and misrepresent the association between exercise and disease severity. Moreover, as HKOA onset and severity is multifactorial (1), several important factors may have not been included in this analysis, namely other psychosocial factors and previous injuries.

### Strengths and Implications

This is the first study with a representative sample of the Portuguese population with HKOA that characterised this population and analysed factors associated with disease severity. Unlike most epidemiologic research on HKOA, the radiographic and clinical severity analyses in this study allow a deeper understanding of people with HKOA from their lived experience of the disease as well as from a structural perspective. Moreover, our case definition of people with HKOA also included people with early-onset (≥18 years old) and early stages of the disease (0–4 grade K-L), allowing more accurate results.

Given the risk factors for HKOA onset and greater severity present in this study's sample and also in the overall Portuguese population (9), our study raises concerns regarding the need for preventive measures and political strategies to improve lifestyle factors and specific interventions directed at people with HKOA. Furthermore, sociodemographic and health-related data from our sample of Portuguese individuals suggest that the socioeconomic and individual burden of this disease may increase over the next decades. Clearly, there is a gap between international recommendations for physical activity and weight management in OA and current care given the small proportion of people who exercise and the large proportion of people who are overweight or obese. Adherence to behavioural strategies and access to care have already been identified as barriers to the optimization of health management among people with HKOA. Nonetheless, health professionals' lack of awareness of OA as a serious disease and lack of knowledge of current recommendations should also be taken into account by health politicians and managers to promote a collective approach to preventing and treating this disease (57).

## Conclusions

Hip and/or Knee OA is present in 14.1% of the Portuguese adults. Age, female sex, multimorbidity, lower education, higher BMI, and anxiety symptoms are associated with higher clinical severity of HKOA, whereas age, geographic location, male sex, and clinical severity are associated with higher radiographic severity of HKOA. Given the cross-sectional design of this study, these factors should be interpreted as an association with higher severity, and not a cause of higher severity. Known risk factors for OA severity, such as decreased physical activity, obesity, and multimorbidity, are highly present among the population of people with HKOA in Portugal. Our findings highlight the need for effective prevention and management strategies focused on identified risk factors, namely weight management and exercise programs and control of chronic non-communicable diseases.

## Data Availability Statement

The data underlying this article were provided by the EpiDoc Unit - CEDOC by permission. Data will be shared upon request to the corresponding author with the permission of EpiDoc Unit group leaders.

## Ethics Statement

The studies involving human participants were reviewed and approved by the Ethics Committee of NOVA Medical School and Portuguese Data Protection Authority (Comissão Nacional de Proteção de Dados). The patients/participants provided their written informed consent to participate in this study.

## Author Contributions

DC and CS contributed to the drafting of the manuscript. DC, EC, CN, and AR contributed to the analysis and interpretation of the data and statistics. HC, JB, and AR contributed to the conception and design of the main project (EpiReumaPt), provision of study materials, obtaining funding for the main project, administrative/logistic support, and collection of data. All authors critically revised and approved the final manuscript.

## Funding

This work was supported by an independent research grant from *Pfizer*. DC received national funding through FCT—Fundação para a Ciência e Tecnologia, I. P. under the Ph.D. grant SFRH/BD/148420/2019.

## Conflict of Interest

This study received funding from an independent research grant by Pfizer. DC received a grant from FCT—Fundação para a Ciência e Tecnologia, I. P. under the Ph.D. grant SFRH/BD/148420/2019. The funders was not involved in the study design, collection, analysis, interpretation of data, the writing of this article or the decision to submit it for publication. The remaining authors declare that the research was conducted in the absence of any commercial or financial relationships that could be construed as a potential conflict of interest.

## Publisher's Note

All claims expressed in this article are solely those of the authors and do not necessarily represent those of their affiliated organizations, or those of the publisher, the editors and the reviewers. Any product that may be evaluated in this article, or claim that may be made by its manufacturer, is not guaranteed or endorsed by the publisher.
