# Supplementary material for: Factors Associated With Clinical and Radiographic Severity in People With Osteoarthritis: A Cross-Sectional Population-Based Study
Source: Front Med (Lausanne). 2021 Nov 15;8:773417. doi: 10.3389/fmed.2021.773417 (PMC8634437; doi:10.3389/fmed.2021.773417)
Supplement: Supplementary file 1 [file Data_Sheet_1.docx]

Supplementary Material

# Supplementary Figures

**Supplementary Figure 1.** HOOS/KOOS subscale scores by sex, age class, and radiographic severity.

# Supplementary Tables

**Supplementary Table 1.**

| Table S1. Sociodemographic, lifestyle, and clinical characteristics of the total sample and the subsample with X-rays | | | |
| --- | --- | --- | --- |
|  | **Total** | **Sample with X-rays** | **p-value^a^** |
| Sample size | n=996 | n=440 |  |
| Age (mean±SD) | 64.39±12.90 | 66.07±10.61 | 0.010 |
| <45 years old | 41 (6.3) | 8 (3.1) | 0.032 |
| 45-54 years old | 146 (16.7) | 48 (16.0) |  |
| 55-64 years old | 289 (22.7) | 112 (21.5) |  |
| 65-74 years old | 368 (31.1) | 157 (32.3) |  |
| ≥75 years old | 243 (23.3) | 115 (27.1) |  |
| Female sex, n (%) | 720 (65.8) | 308 (67.6) | 0.181 |
| Geographic location, n (%) |  |  | <0.001 |
| North | 271 (35.8) | 190 (45.4) |  |
| Centre | 243 (27.7) | 109 (22.1) |  |
| Alentejo | 67 (6.5) | 25 (4.6) |  |
| Algarve | 21 (1.8) | 6 (0.9) |  |
| Lisbon | 162 (23.5) | 88 (26.1) |  |
| Islands | 231 (4.7) | 22 (0.8) |  |
| Marital status (partner), n (%) | 639 (64.4) | 302 (69.8) | 0.009 |
| Education level, n (%) |  |  | 0.282 |
| <4 years | 246 (22.6) | 121 (25.0) |  |
| 4-9 years | 631 (62.9) | 275 (61.8) |  |
| ≥10 years | 117 (14.4) | 43 (13.1) |  |
| BMI, n (%) |  |  | 0.156 |
| Underweight | 3 (0.2) | - |  |
| Normal weight | 162 (20.5) | 65 (17.4) |  |
| Overweight | 387 (43.6) | 172 (45.9) |  |
| Obese | 369 (35.8) | 167 (36.6) |  |
| Lifestyle variables, n (%) |  |  |  |
| Smoker | 71 (10.7) | 22 (6.7) | 0.016 |
| Alcohol consumption (daily) | 480 (54.4) | 232 (56.6) | 0.483 |
| Regular exercise | 209 (21.3) | 86 (18.8) | 0.087 |
| Clinical variables |  |  |  |
| HOOS/KOOS, mean±SD  (min-max) | 55.79±20.88  (0.00-100) | 51.29±19.11  (0.00-100) | <0.001 |
| HOOS/KOOS  low tertile, n (%) | 281 (33.8) | 88 (26.5) | 0.004 |
| HOOS/KOOS  middle tertile, n (%) | 361 (32.8) | 131 (33.8) |  |
| HOOS/KOOS  high tertile, n (%) | 354 (33.4) | 169 (39.7) |  |
| Number of non-communicable diseases, mean±SD | 2.91±1.96 | 3.10±1.99 | 0.011 |
| Anxiety (HADS-A), mean±SD | 6.70±4.21 | 6.96±4.18 | 0.137 |
| With anxiety symptoms (HADS-A≥11), n (%) | 193 (18.5) | 87 (18.9) | 0.553 |
| Depression (HADS-D), mean±SD | 6.04±4.49 | 5.62±4.14 | 0.097 |
| With depression symptoms (HADS-D≥11), n(%) | 159 (16.8) | 82 (19.3) | 0.043 |
| ^a^ Significance level of independency test between the subsamples with and without X-rays: complex samples t-test for continuous variables and Chi-square tests for categorical variables. All percentages and mean±SD were weighted | | | |

**Supplementary Table 2.**

| Table S2: Sociodemographic and anthropometric, clinical and lifestyle characteristics of participants with Knee and Hip OA | | |
| --- | --- | --- |
|  | Hip OA | Knee OA |
| Sample size | n=199 | n=981 |
| Age (mean±SD) | 63.86±12.81 | 66.62±12.98 |
| <45 years old, n (%) | 10 (6.7) | 33 (6.2) |
| 45-54 years old, n (%) | 22 (17.7) | 133 (15.6) |
| 55-64 years old, n (%) | 37 (21.4) | 270 (22.9) |
| 65-74 years old, n (%) | 77 (31.0) | 327 (31.3) |
| ≥75 years old, n (%) | 53 (23.2) | 218 (23.9) |
| Female, n (%) | 119 (53.3) | 716 (67.0) |
| Geographic location, n (%) |  |  |
| North | 60 (37.9) | 256 (33.9) |
| Centre | 50 (29.4) | 244 (27.9) |
| Lisbon | 42 (22.4) | 25.1 (163) |
| Alentejo | 14 (6.1) | 65 (6.6) |
| Algarve | 3 (1.5) | 19 (1.7) |
| Islands | 30 (2.8) | 234 (4.8) |
| Marital status (partner), n (%) | 135 (68.7) | 622 (63.6) |
| Years of education, n (%) |  |  |
| <4 years | 47(18.3) | 247 (23.5) |
| 4-9 years | 124 (70) | 618 (60.9) |
| ≥10 years | 27 (11.8) | 115 (15.6) |
| BMI, n (%) |  |  |
| Underweight | 1 (0.6) | 2 (0.1) |
| Normal weight | 41 (27.6) | 156 (19.2) |
| Overweight | 73 (41.8) | 381 (43.4) |
| Obese | 68 (30) | 370 (37.3) |
| Lifestyle variables, n (%) |  |  |
| Smoker | 10 (10.3) | 71 (9.9) |
| Alcohol intake (daily) | 61 (34.8) | 200 (26.9) |
| Regular exercise | 41 (18.4) | 202 (21.2) |
| Clinical variables, mean±SD |  |  |
| HOOS/KOOS  (min-max) | 59.46±21.78  (4-100) | 55.33±20.64  (0-100) |
| Multimorbidity (yes), n (%) | 149 (71.9) | 747 (74.0) |
| Anxiety symptoms (HADS-A), mean±SD | 6.49±4.30 | 6.65±4.15 |
| HADS-A≥11, n (%) | 35 (17.1) | 188 (17.8) |
| Depression symptoms (HADS-D), mean±SD | 5.74±4.30 | 5.98±4.49 |
| HADS-D≥11, n (%) | 31 (12.7) | 152 (16.4) |

**Supplementary Table 3**

| Table S3. Factors associated with clinical severity in univariate ordinal regression analysis | | |
| --- | --- | --- |
|  | **OR (95% CI)** | **p-value** |
| Age class |  | <0.001 |
| <55 years old^a^ | - | - |
| 55-64 years old | 3.27 (1.81, 5.95) | <0.001 |
| 65-74 years old | 2.31 (2.56, 7.29) | 0.001 |
| ≥75 years old | 6.06 (4.33, 1.00) | <0.001 |
| Sex | |  |
| Male^a^ | - | - |
| Female | 2.29 (1.53, 3.42) | <0.001 |
| Chronic non-communicable diseases |  |  |
| No multimorbiditiy^a^ | - | - |
| Multimorbidity | 2.90 (1.90, 4.42) | <0.001 |
| Geographic location |  | 0.120 |
| North^a^ | - | - |
| Center | 1.19 (0.52, 2.87) | 0.472 |
| Lisbon | 0.87 (0.52, 1.44) | 0.583 |
| Alentejo | 1.70 (1.04, 2.78) | 0.034 |
| Algarve | 1.09 (0.38, 3.08) | 0.875 |
| Islands | 0.90 (0.60, 1.36) | 0.616 |
| Marital status |  |  |
| Without partner^a^ | - | - |
| With partner | 1.165 (0.79, 1.70) | 0.430 |
| Education level |  | <0.001 |
| <4 years^a^ | - | - |
| 4-9 years | 0.31 (0.22, 0.45) | <0.001 |
| ≥10 years | 0.15 (0.09, 0.27) | <0.001 |
| BMI (kg‎/m^2^) |  | 0.002 |
| Normal or underweight (<25kg/m^2^)^a^ |  |  |
| Overweight (25-29.99 kg‎/m^2^) | 1.87 (1.06, 3.30) | 0.003 |
| Obese (≥30 kg‎/m^2^) | 2.72 (1.53, 4.85) | 0.001 |
| Anxiety (HADS-A) |  |  |
| No anxiety symptoms (HADS-A<11)^a^ | - | - |
| Anxiety symptoms (HADS-A≥11) | 1.96 (1.33, 2.80) | 0.001 |
| Depression (HADS-D) |  |  |
| No Depression symptoms (HADS-D<11)^a^ | - | - |
| Depression Symptoms (HADS-D≥11) | 1.94 (1.66, 3.22) | 0.011 |
| Smoking habits |  |  |
| Non-smoker^a^ | - | - |
| Daily or occasional smoker | 0.38 (0.19, 0.75) | 0.005 |
| Alcohol consumption |  |  |
| Never or occasionally^a^ | - | - |
| Daily | 0.67 (0.44, 1.03) | 0.065 |
| Exercise |  |  |
| No regular exercise^a^ | - | - |
| Regular exercise | 0.52 (0.34, 0.80) | 0.003 |
| ^a^Reference classes  All analyses were weighted. | | |

**Supplementary Table 4.**

| Table S4. Factors associated with radiographic severity in univariate ordinal regression analysis | | |
| --- | --- | --- |
|  | **OR (95% CI)** | **p-value** |
| Age |  | 0.020 |
| <55 years old^a^ | - | - |
| 55-64 years old | 1.37 (0.57, 3.26) | 0.478 |
| 65-74 years old | 2.60 (1.18, 5.71) | 0.018 |
| ≥75 years old | 2.71 (1.16, 6.35) | 0.022 |
| Sex |  |  |
| Male^a^ | - | - |
| Female | 0.65 (0.42, 1.00) | 0.052 |
| Chronic non-communicable diseases |  |  |
| No multimorbiditiy^a^ |  |  |
| Multimorbidity | 1.43 (0.81, 2.55) | 0.220 |
| Geographic location |  | 0.003 |
| North^a^ | - | - |
| Center | 0.49 (0.29, 0.83) | 0.008 |
| Lisbon | 0.30 (0.16, 0.57) | <0.001 |
| Alentejo | 0.90 (0.46, 1.78) | 0.770 |
| Algarve | 0.92 (0.25, 3.45) | 0.899 |
| Islands | 0.49 (0.21, 1.15) | 0.099 |
| Marital status | - | - |
| Without partner^a^ | - | - |
| With partner | 0.91 (0.56, 1.48) | 0.699 |
| Years of education |  | 0.001 |
| <4 years^a^ |  |  |
| 4-9 years | 0.48 (0.30, 0.75) | 0.002 |
| ≥10 years | 0.17 (0.07, 0.45) | <0.001 |
| BMI (kg‎/m^2^) |  | 0.136 |
| Normal or underweight (<25kg/m^2^)^a^ | - | - |
| Overweight (25-29.99 kg‎/m^2^) | 0.27 (1.77, 1.59) | 0.270 |
| Obese (≥30 kg‎/m^2^) | 2.06 (0.97, 4.45) | 0.065 |
| HOOS/KOOS |  | <0.001 |
| Low tertile^a^ | - | - |
| Middle tertile | 1.40 (0.70, 2.94) | 0.370 |
| High tertile | 3.68 (1.82, 7.43) | <0.001 |
| Anxiety (HADS-A) |  |  |
| No anxiety symptoms (HADS-A<11)^a^ | - | - |
| Anxiety symptoms (HADS-A≥11) | 1.10 (0.64, 1.89) | 0.738 |
| Depression (HADS-D) |  |  |
| No depression symptoms (HADS-D<11)^a^ | - | - |
| Depression symptoms (HADS-D≥11) | 0.94 (0.48, 1.83) | 0.845 |
| Smoking habits |  |  |
| Non-smoker^a^ | - | - |
| Daily or occasional smoker | 0.44 (0.17, 1.16) | 0.097 |
| Alcohol consumption |  |  |
| Never or occasionally^a^ | - | - |
| Daily | 1.25 (0.77, 2.04) | 0.369 |
| Exercise |  |  |
| No regular exercise^a^ | - | - |
| Regular exercise | 0.49 (0.27, 0.87) | 0.016 |
| ^a^Reference classes  All analysis were weighted. | | |
