# Supplementary figures and images for: Factors Associated With Clinical and Radiographic Severity in People With Osteoarthritis: A Cross-Sectional Population-Based Study
Source: Front Med (Lausanne). 2021 Nov 15;8:773417. doi: 10.3389/fmed.2021.773417 (PMC8634437; doi:10.3389/fmed.2021.773417)

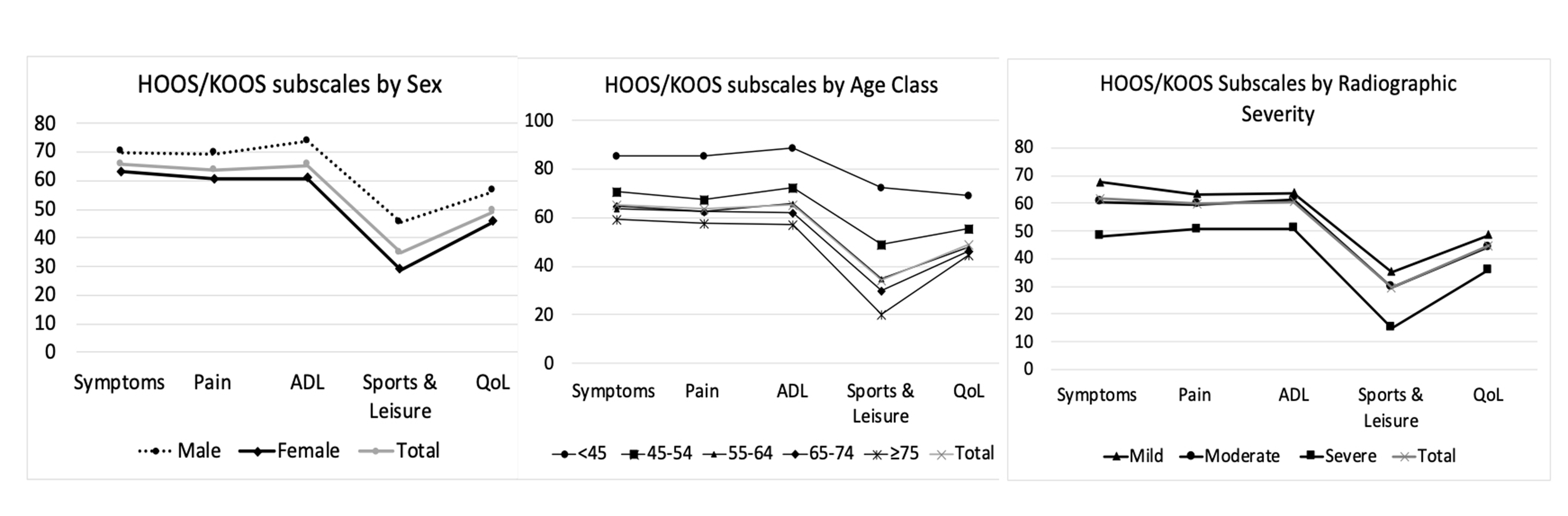

Supplement: Supplementary file 2 [file Image_1.JPEG]
